# Building a ‘Virtual Library’: continuing a global collaboration to strengthen research capacity within Nepal and other low- and middle-income countries

**DOI:** 10.1080/16549716.2022.2112415

**Published:** 2022-10-06

**Authors:** Catherine E. Elmore, Sandhya Chapagain Acharya, Soniya Dulal, Flannery Enneking-Norton, Pawan Kumar Hamal, Regina Kattel, Martha A. Maurer, Damodar Paudel, Bishnu Dutta Paudel, Ramila Shilpakar, Deepak Sundar Shrestha, Usha Thapa, Daniel T. Wilson, Virginia LeBaron

**Affiliations:** a University of Virginia School of Nursing, Charlottesville, Virginia, USA; b National Academy of Medical Sciences, Bir Hospital, Kathmandu, Nepal; c Department of Internal Medicine, B.P. Koirala Institute of Health Sciences, Dharan, Nepal; d National Academy of Medical Sciences, National Trauma Center, Kathmandu, Nepal; e Department of Palliative Medicine, Nepal Cancer Hospital and Research Center, Patan, Nepal; f University of Wisconsin - Madison School of Pharmacy, Sonderegger Research Center, Madison, Wisconsin, USA; g Department of Medicine, Nepal Police Hospital, Kathmandu, Nepal; h Department of Medical Oncology, Bhaktapur Cancer Hospital, Bhaktapur, Nepal; i Peoples Dental College and Hospital, Kathmandu, Nepal; j B.P. Koirala Memorial Cancer Hospital, Bharatpur, Nepal; k Claude Moore Health Sciences Library, University of Virginia, Charlottesville, Virginia, USA

**Keywords:** Research capacity building, non-communicable diseases, low- and middle-income countries, community based participatory research, digital library

## Abstract

To fill the gap in health research capacity-building efforts, we created the ‘Virtual Library’ (VL) – a web-based repository of context-relevant resources for health researchers in low- and middle-income countries (LMICs). This paper describes the participatory process used to systematically develop the VL, and describes how our interprofessional team – representing both an LMIC (Nepal) and a high-income country (HIC) (USA, US) – engaged in shared meaning-making. A team of researchers and clinicians representing a range of subdisciplines from Nepal and the US created a replicable search strategy and standardized Resource Screening Guide (RSG) to systematically assess resources to be included within the VL. Descriptive methods were used to summarize findings from the RSG and lessons learned from the collaborative process. Collectively, 14 team members reviewed 564 potential resources (mean = 40, SD = 22.7). Mean RSG score was 7.02/10 (SD = 2). More than 76% of resources met each of the four quality criteria (relevant; reputable, accessible; understandable). Within the published VL, 298 resources were included, organized by 15 topics and 45 sub-topics. Of these, 223 resources were evaluated by the RSG; 75 were identified by team member expertise. The collaborative process involved regular meetings, iterative document revisions, and peer review. Resource quality was better than expected, perhaps because best practices/principles related to health research are universally relevant, regardless of context. While the RSG was essential to systematize our search and ensure reproducibility, team member expertise was valuable. Pairing team members during peer-review led to bi-directional knowledge sharing and was particularly successful. This work reflects a highly collaborative global partnership and offers a model for future health research capacity-building efforts. We invite engagement with the Virtual Library <https://lmicresearch.org> as one supportive pillar of infrastructure to develop individual and institutional research capacity.

## Purpose

Health research capacity building (HRCB) is the process by which institutions, and the individuals who work within them, improve their ability to develop, implement, and sustain high-quality research efforts which can be translated into practice in order to improve health outcomes [1,2]. HRCB, also known as capacity strengthening or capacity development [3], is not only vital in low- and middle-income countries (LMICs) for the fight against non-communicable diseases (NCD) including cancer, but is also a holistic approach to develop research capacity in all domains of public health response [4–7]. Both the World Health Organization and the United Nations have identified HRCB in LMICs as a priority objective for the prevention and control of NCDs [8,9].

Repositories of web-based, open-access resources to help researchers and clinicians conduct research have the potential to support HRCB in limited-resource contexts. While there are many descriptive papers about how HRCB efforts have been implemented across global contexts [10–25] and a wide range of diseases and population foci [10,12,14–16,18–23,25–28], there are few examples of HRCB resource repositories. Those that do exist appear poorly organized, are more than 10 years old, include non-functional links, or lack a specific focus on the needs of novice researchers in LMICs or clarity regarding how resources were selected for inclusion [28–32]. Furthermore, discovery of these repositories can be challenging, often most likely identified by hand-searching the reference lists of published HRCB projects. Importantly, we found that existing repositories lacked an explicitly collaborative approach for systematic identification and evaluation of resources and the co-creation of the published repository by team members from both LMIC and HIC research partners.

Our team sought to fill this gap by collaboratively creating a dynamic, web-based, open-access repository – called the ‘Virtual Library’ (VL) – organized by stages of the research process and designed to support novice researchers in limited-resource settings. The purpose of this paper is two-fold: to describe the systematic development of the VL, as well as the process by which our global team – representing both a LMIC (Nepal) and a HIC (USA, US) – engaged in shared meaning-making, which is essential to global collaborations [33]. To our knowledge, this is the first paper that details such an effort; our process can be used as a model for other collaborative HCRB efforts.

## Methods

This article represents one outcome of a larger research collaboration between the Nepalese Association of Palliative Care (‘NAPCare’) and the University of Virginia (UVA; in the US), which evolved from partnerships first formed between Nepal and US investigators in 2004. NAPCare is a non-profit, non-governmental organization founded in 2009 to advocate for palliative care within Nepal [34]. The primary aim of the NAPCare-UVA collaboration, consistent with the overall objective of the project’s funding mechanism [35], is to strengthen individual and institutional research capacity related to cancer care within Nepal. This aim was achieved by conducting a comprehensive survey of cancer care institutions within Nepal [36]; developing and pilot testing a mobile application to support healthcare providers in delivering evidence-based cancer pain management [37]; and creation of a ‘Virtual Library,’ (VL) which is the focus of this paper.

### Project design

**Figure 1.**
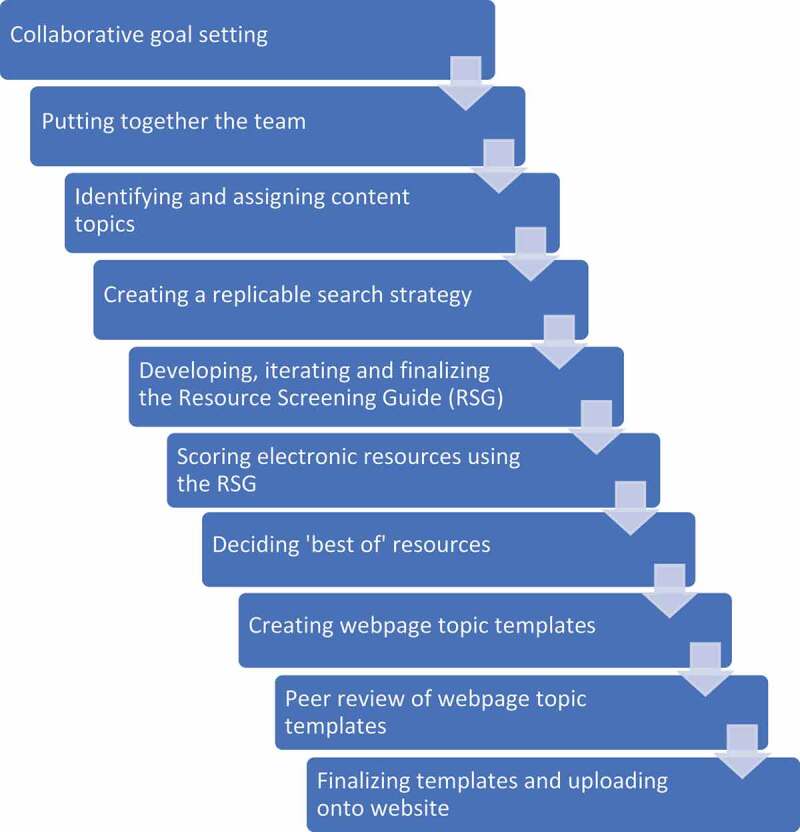
Overview of the process for creating the Virtual Library.

The overall goal for the VL was to utilize a highly collaborative, participatory process [24] to systematically identify contextually and culturally relevant resources to facilitate independent clinical research in LMICs and organize them in a user-friendly, web-based, open-access platform. This paper describes the process (Figure 1) and outcomes of creating the VL, including: (1) creation of a replicable search strategy for identifying resources; (2) development of a standardized screening guide to assess resource quality; (3) results of resource screening; and (4) lessons learned from engaging in this collaborative HRCB project.

This figure shows an overview of the step by step process our global interprofessional team used to develop the Virtual Library. Each step is described in detail in the manuscript. RSG = Resource screening guide.

### Participants & setting

All members of the collaboration were invited to participate in creating the VL by the NAPCare-UVA PIs. Thus, the VL team initially consisted of self-selected individuals from both Nepal and UVA, who were concurrently working on other aspects of the larger NAPCare-UVA project and who expressed an interest in being part of the VL work. After the initial meetings, recognizing the need for additional expertise, team members extended personal invitations to others who would be equally dedicated and committed to the work. Specifically, Nepalese team members (physicians and nurses, clinicians and faculty in both medicine and nursing) invited four other Nepalese clinician colleagues known to have a strong research background, and whose participation helped to increase the diversity of institutional affiliations both in and out of the Kathmandu Valley, and broaden the representation of clinical subspecialities (e.g. medicine, anesthesia, palliative care, oncology). This group included current or former members of the Nepal Health Research Council (NHRC), a governmental organization responsible for high-quality, ethical health research in Nepal [38], as well as the editor of the Journal of the NHRC. UVA team members (PhD-trained nursing and social work researchers with clinical expertise in oncology, palliative care, emergency care, and rural/urban primary care) extended invitations to a website developer, a health sciences librarian, and four undergraduate nursing students enrolled in a research practicum. The final VL project team equally represented both Nepal and UVA.

Creating the VL was a highly iterative process that occurred over a 2-year period (2019–2021), starting with an initial in-person brainstorming meeting which was held in Nepal in January 2019. Overall coordination of the VL project was led by UVA and Nepal principal investigators (PIs) and facilitated by group emails, collaborative use of Google Drive applications (including Docs, Sheets and Forms), and regular meetings using video conferencing (Zoom). Between October 2020 and April 2021, we held 19 biweekly 1-hour-long Zoom meetings, which were recorded and made available for all team members who were unable to attend. Additional in-person meetings were planned, but had to be cancelled because of the COVID-19 global pandemic.

### Identifying content topics

Using the research process as a framework, we sketched out an initial list of specific topics to be included. For each topic, we created a guiding question (Table 1), to focus the search.

**Table 1.** Virtual library topic, guiding question, and search query for a sample of selected topics.

*Topic*: An Introduction to Clinical Research: The Research Process and Goals*Guiding Question*: Why do we conduct clinical research and how do we create a plan to complete the research?*Search Query*: (introduction OR basics) AND (‘clinical trials’ OR ‘clinical research’)
*Topic*: Reviewing the Literature*Guiding Question*: How to understand what is known about your research topic and what research is needed.*Search Query*: (introduction OR ‘how to’) AND ‘literature review’
*Topic*: Ethical Issues/Considerations: Overview*Guiding Question*: What ethical issues are important to consider when we conduct human subject research?*Search Query*: (ethical OR ethics) AND (‘human subjects research’ OR ‘human research’)

A key goal was that the topics be pertinent for beginning clinical researchers in Nepal and similar limited-resource contexts. Our target audience is not necessarily related to an academic role, degree, or particular profession; but rather, anyone who is new to the field of clinical research. This might include a nurse who has not had training in research but wants to become more involved, a physician who had research training in the distant past and wants to refresh their skills, or a new PhD student. Further, we added, subtracted and modified topics based on discussions about relevance to novice researchers or LMIC contexts. Special topics (e.g. ‘Mobile Health’, ‘NCD Research’) were included due to their relevance to the parent project aims and LMICs in general.

Team members signed up for topics (~2-5 each) and claimed responsibility for identifying content for the corresponding VL webpage. In this way, individuals developed ‘ownership’ and were considered the ‘expert’ for that content area.

### Resource screening guide development & creating a replicable search strategy

Four foundational quality criteria were used to evaluate potential content: relevant, reputable, accessible, and understandable. We arrived at these final four quality criteria after extensive group discussion about goals and priorities for the VL. In collaboration with the team’s health sciences librarian, we also conducted exploratory searches for potentially relevant resources, which allowed us to discuss, iterate, and achieve group consensus on how to operationalize these criteria into screening questions. Further, we found support for these key concepts in the literature on best practices for digital repositories [39–44].

We created a Resource Screening Guide (RSG; shown in Table 2) to systematically assess how well each potential resource met the four quality criteria using a simple point-system. We hypothesized that resources with preferred qualities would score higher, facilitating more objective decisions about which resources should ultimately be included in the VL.

**Table 2.** Final version of the Virtual Library resource screening guide (using google forms, with the ‘quiz’ feature).

**Instructions**: This guide screens resources on four criteria: Relevant, Reputable, Accessible, and Understandable. The guide scores resources on an 8 (if not a video) or 10 (if video) point scale (0, worst; 8/10, best). The guide also asks the reviewer to give their subjective opinion of whether they think the resource should be included in the Virtual Library, regardless of how it scores.	
Questions	Point Value
1. Name of reviewer (select from dropdown)	
2. What is the Virtual Library topic category for this resource? (select from dropdown; 47 choices including ‘Other/unsure’)	
3. Title of Resource (cut and paste exact title)	
4. URL of Resource (cut and paste exact web address)	
5. Did you change/add any search terms from the suggested search strategy? (yes/no) a. If yes, please record your revised/updated search terms here (copy and paste the new search).	
6. Which database did you search? (GCS, YouTube, Google)	
7. Video details (if found on YouTube) a. Is the creator/originator of the video reputable? (for example, the creator’s website ends in .gov, .edu, .np or .org) b. How long is the video? c. Are accurate ‘closed captions’ available?	1 if Yes 1 if Yes
**Relevant**	
8. Is the resource useful to conduct research in Nepal (or similar settings)?	1 if Yes
9. Which statements are TRUE about the resource? (check all that apply) a. The resource uses multiple examples or references specific to Nepal or similar countries. b. The resource uses multiple examples or references specific to the U.S. or similar countries. c. The resource assumes access to advanced resources or technology. d. The research is about best practices or core research principles that could apply to any setting. e. Other/None of the above f. If you selected ‘Other/None of the above,’ please describe the resource and its relevance to Nepal/LMICs in your own words.	
**Reputable**	
10. Is the content current (updated within past 5–7 years)?	1 if Yes
11. Is there potential bias, commercial, or political interest?	1 if No
**Accessible**	
12. Is an account required to access content?	1 if No
13. Is payment required to access resource?	1 if No
**Understandable**	
14. Is the resource helpful and useful to a beginner or new researcher? (for example, is it a primer, introduction, overview, or a ‘how-to’ guide?)	1 if Yes
15. Is the language clear and simple? (for example, not too technical or complicated)?	1 if Yes
**Reviewer Recommendation**	
16. What sub-category of the website does this resource belong in?	
17. How easy was it for you to review this resource?	
18. In your opinion, should this resource be included in the Virtual Library?	1 if Yes
19. Please record any questions, comments, or concerns about this resource.	

We used Google Forms (using the ‘quiz’ option for points) for the RSG. Google Forms was preferred over another platform due to accessibility (cost and institutional access) and ease of use for data collection and analysis. Because we were concerned about reviewer burden, we were interested in tracking how long it took team members to review one resource. Google Form lacks this time-to-completion feature, but we overcame this by adding a question ‘How easy was it for you to review this resource?’ to the RSG. An initial RSG was iterated and revised in pilot testing, then all members were trained in its use.

The librarian identified appropriate search databases that are accessible in LMICs and would provide results across a range of content. Three databases were tested initially: (1) Pub Med Central (PMC); (2) Google Custom Search (GCS); and (3) YouTube.

PMC was chosen as a reputable, open-access database for searching published, peer-reviewed, scientific literature. Including open-access peer-reviewed literature (e.g. which does not require institutional subscriptions or fees for accessing full-text content) was a key priority.

GCS is a programmable search engine [45] that offered reach into the grey literature [46], which, while not necessarily peer-reviewed, provided valuable results when carefully filtered. The librarian programmed a customized search to retrieve results primarily from .gov, .edu, and .org sites. Given the global scope of this project, GCS settings were established so that the search would include international domains (e.g. <.gov.np> from Nepal and <.gov.in> from India), while excluding domains ending in <.com> in order to avoid commercial, for-profit sources.

YouTube allowed identification of resources more accessible to learners who benefit from audio/visual content. Through team discussion, we opted for YouTube presentations developed by individuals associated with academic institutions or professional societies versus content produced by for-profit companies.

Initial search queries for each topic (Table 1) were designed by the librarian, tested using Boolean operators (e.g. and/or/not) and keywords, and vetted by team members. Initially, the same search query was used consistently across databases. Given that default search algorithms sort by relevance, and vary based on IP address location (e.g. we found top results displayed differed slightly in Nepal versus the US), we agreed to focus on the top 10 results (excluding advertisements), but explicitly allowed flexibility to review more/fewer, based on individual judgment.

After an initial round of searching and discussion, we made four changes to the RSG: (1) added an option to revise search queries; (2) removed PMC as a database because language used by the resources was too technical, articles often focused on highly specialized topics, and did not seem appropriate for beginning researchers; (3) added an option to search with Google versus GCS; and (4) added two summary subjective questions, ‘*How easy was it for you to review this resource*?’ and ‘*In your opinion, should this resource be included in the VL?*’. The final RSG is shown in Table 2.

### Finalizing content

Once searching and screening was complete, each member was given RSG data for the topics they searched. Team members then individually reviewed their data, revisited resources, and re-considered quality criteria and scoring. Team discussion about how to select the final content centered around growing collective awareness that as we conducted searches and evaluated potential resources, our understanding of the ‘best’ resources was evolving. Instead of relying solely on the RSG score (choosing high-scoring resources), we also considered the reviewer’s expert opinion, which was more difficult to quantify. This decision-making process is conceptualized in Table 3, which shows how we considered the RSG’s objective scoring alongside our informed subjectivity. Finally, our goal was quality over quantity: we aimed to create a smaller list of highly curated resources, versus a longer list of potentially less helpful resources.

**Table 3.** Guiding heuristic describing how team members could appraise the objective scoring of the Resource Screening Guide (RSG) against their subjective judgment to decide what to include in the Virtual Library.

Subjective Decision vs. Objective Score	High RSG Score +	Low RSG Score -
Yes, recommend inclusion in VL +	Include +/+	Maybe +/-
No, do not recommend inclusion in VL -	Maybe -/+	Exclude -/-

Once the ‘best’ resources were selected, team members used a standardized web-page template to develop draft webpages for their topics. The purpose of the template was to ensure overall consistency in navigation across all pages of the website and to simplify the upload of content by the website developer. The template was only modified if the original template creator did not identify a high-quality resource for a particular sub-heading. For example, if there were no high-quality ‘On-line Courses’, then this sub-heading was removed. The web-page template is included in the (Table S6).

### Peer review of finalized content

Draft webpage templates were saved in a shared Google Drive folder and exchanged for peer review. The peer review process was purposely designed so that templates created by UVA team members were reviewed by Nepalese team members and vice versa, with a key goal to ensure identified content was perceived as appropriate and relevant by global counterpart team members. Each person was assigned 2–3 templates for peer review, and were invited to review any other template(s) of interest. Peers examined the resources in light of the four quality criteria, added comments, and proposed suggestions. Once peer review was complete, the original template creator reviewed their peers’ feedback, made revisions, and finalized the content. Finally, each finalized template went through a final formatting checklist (Table 7 found in the Online Supplementary Material) before it was sent to the website developer.

### Data collection & analysis

Quantitative data from the RSG were exported from Google Forms. After removing duplicate URLs, we generated descriptive statistics using Microsoft Excel. Qualitative data were collected throughout the project, including meeting and audit trail notes. We re-read notes to summarize salient aspects of the process, reflected on lessons learned, and created shared meaning around data interpretation and process evaluation.

## Results

The VL website was designed in Word Press and hosted by Amazon Web Services. The final structure, aesthetics, and navigation/organization were collaboratively reviewed and iterated by the entire global, interprofessional team.

### Results of the RSG

Summary statistics related to the RSG are found in Table 4. We evaluated a total of 564 unique resources, with 14 team members reviewing an average of 40 potential resources (SD = 22.7). The mean RSG score was 7.02 (SD = 2) out of 10. Over a third of resources were found using GCS (40.3%); 35.6% from YouTube, and 24.1% from Google. Once given the option to revise/change search queries, team members indicated they did so in 39.2% (*n* = 159 of 406) of searches. Examples of revised search queries included those used to identify known international standards in research (e.g. ‘Cochrane’, ‘the PRISMA statement’).

**Table 4.** Summary of resource evaluation guide scores.

Overall Screening	Frequency (Percent)	Mean Score (out of 10)	
Total resources screened by RSG	564 (100%)	7.02	
**Resources Screened per Database**			
Google Custom Search	227 (40.3%)	6.43	
YouTube	201 (35.6%)	7.86	
Google*	136 (24.1%)	7.46	
**Video details (*n* = 201)**			
	**‘Yes’**	**‘No’**	**‘Unsure’**
Reputable creator	73 (36.3%)	98 (48.8%)	30 (14.9%)
Closed Captions available	114 (56.7%)	74 (36.8%)	13 (6.5%)
	**<15**	**16–30**	**31+**
Length of video (count by minutes)	143 (71.1%)	33 (16.4%)	25 (12.4%)
**Quality Criteria Details**			
**Relevant**	**‘Yes’**	**‘No’ or ‘Unsure’**	
Useful in Nepal/LMIC	437 (77.5%)	127 (22.5%)	
Which statements are true about the resource? (Select all that apply)			Frequency
Focuses on best practices/core principle			54
References U.S./Western context			15
Assumes access to advanced resources or technology			8
Other reason**			22
**Reputable**	**‘Yes’**	**‘No’**	**‘Maybe’ or ‘Unsure’**
Current (5–7 years)	431 (76.4%)	78 (13.8%)	55 (9.8%)
Potential bias	28 (4.9%)	455 (80.7%)	81 (14.4%)
**Accessible**	**‘Yes’**	**‘No’**	
Account required	24 (4.3%)	540 (95.7%)	
Payment required	19 (3.4%)	545 (96.6%)	
**Understandable**	**‘Yes’**	**Unsure**	**No**
Useful to beginner	476 (84.4%)	57 (10.1%)	31 (5.5%)
Language clear and simple	488 (86.5%)	53 (9.4%)	23 (4.1%)
**Subjective Reviewer Feedback**			
	**Very Easy/Easy**	**Neutral**	**Difficult/Very Difficult**
Ease of review	390 (69.2%)	146 (25.9%)	27 (4.9%)
	**‘Yes’**	**‘No’**	**‘Maybe’ or ‘Review with team’**
In your opinion, should this resource be included in the Virtual Library?	392 (67.2%)	74 (3.1%)	112 (19.9%)

RSG = Resource Screening Guide. LMIC = Low- and middle-income countries.

*Google was not used after the RSG was iterated by the team.

**Open response/free text question. ‘Other reasons’ (*n* = 22) given for a resource not being relevant were most often related to being aimed at a specific general audience (e.g. students attending a specific school, patients and families, consumers of products) and not for health researchers.

In the assessment of resource quality, 77.5% (*n* = 437) were found to be relevant for health research in Nepal/LMICs. Most ‘other reasons’ (*n* = 22) given for a resource not being relevant included being aimed at an unrelated audience (e.g. students attending a specific school, patients/families, consumers of products). 76.4% (*n* = 431) were published within the last 5–7 years; and 80.7% (*n* = 455) appeared free of obvious potential bias (i.e. promoting a commercial product). More than 95% of resources did not require an account or payment for access. More than 84% appeared to be useful to beginner/novice researchers, and used clear, simple language. Of the 201 videos screened, only 36.3% (*n* = 73) seemed to have a reputable creator, while 56.7% (*n* = 114) had accurate closed captioning. The majority of videos (71.1%, *n* = 143) were less than 15 minutes long; 26.4% (n = 53) were less than 5 minutes long.

Over two-thirds of resources (*n* = 390, 69.2%) were reported as ‘easy’ or ‘very easy’ to review. Finally, subjective assessment of individual resources (e.g. ‘In your opinion, should this resource be included in the VL?’) suggested that two-thirds (*n* = 379, 67.2%) should be included in the published VL.

### Content topics and features

Within the published VL, 298 unique peer-reviewed resources were ultimately included, organized under 15 topics and 45 sub-topics (Table 5). This included 223 (40% of 564 total) resources first evaluated by the RSG, plus an additional 75 that were identified by team members as high-quality, well-known resources, such as the World Health Organization (www.who.int), Cochrane database (https://Cochrane.org), and the Directory of Open Access Journals (doaj.org).

**Table 5.** Virtual Library site map with counts of resources screened versus included.

Topics and sub-topics	Number of resources screened using RSG (*n* = 564)	Number of peer-reviewed resources included in VL (n = 298)
Research Basics		
Introduction to Clinical Research		
The Research Process and Goals	20	5
Initial Steps		
Developing a Research Question	10	6
Putting Together Your Team	10	5
Reviewing the Literature	15	12
Choosing a Study Design and Approach		
Overview of Research Study Designs	9	5
Community Based Participatory Research	16	6
Meta-Analysis/Systematic Review	9	7
Qualitative		
Overview of Qualitative Research	18	9
Case Reports	5	4
Ethnography	20	4
Quantitative		
Overview of Quantitative Research	19	7
Experimental/Intervention Studies		
Overview of Experimental Research	3	6
Clinical Trials	8	9
Randomized Controlled Trials	13	5
Observational Studies		
Overview of Observational Research*	–	10
Case Control Studies	19	8
Cohort Studies	21	10
Cross-sectional Studies	22	10
Ethical Considerations		
Overview of Ethical Considerations	24	5
Institutional Review Boards	14	8
Nepal Health Research Council^	–	–
Writing a Research Protocol	6	4
Data Analysis		
Qualitative Data Analysis	18	6
Quantitative Data Analysis	14	5
Sharing Your Results		
Overview of Sharing Results	16	5
Publishing in Academic Journals		
Selecting a Journal	27	9
International and Ethical Standards for Authors	20	9
Writing a Cover Letter	10	6
Preparing and Submitting a Manuscript	10	8
Preparing the Response to Reviewers/Responding to Feedback	10	9
Scholarly Presentations		
Selecting the Conference/Venue	12	7
How to Prepare and Submit an Abstract	17	6
Preparing a Poster	17	8
Preparing an Oral or Podium Presentation	26	10
Special Topics		
Implementation Science	5	5
Mobile Health (‘mHealth’)	20	19
Non-Communicable Disease Research	5	4
Securing Funding		
Funding Options	8	7
Preparing and Submitting the Application	9	11
Grant/Project Management	2	10
Research Capacity Building		
Overview of Research Capacity Building	17	6
The NAPCare-UVA Collaboration^	–	–
Other Examples*	–	3
Other Helpful Resources^	–	–

RSG = Resource Screening Guide

^These resources were added based on recommendation of team members and other expert opinion, and thus were not put through the RSG.

*These categories were not on the original topic list, but emerged after the resource search was completed as team members were creating the webpage templates.

The final VL also contained project-specific webpages (e.g. pages about NHRC and the NAPCare-UVA Collaboration) that did not go through the peer review process. Additional VL features include an ‘About Us’ page describing the project history and purpose; and a ‘Contact Us’ page which allows users to submit questions, feedback, and suggestions. On each page, users are given the option to rate the helpfulness of resources using a 5-star scale. A page called ‘Other Helpful Resources’ includes a list of research databases accessible to individuals and institutions in LMICs (e.g. Hinari Access to Research for Health Programme), plus scholarly writing and global health research resources that we found particularly comprehensive or helpful. Users also have the option to create a VL user account. Our transparent, well-documented search and evaluation process ensures that in the future, seasoned or new team members could apply the same search queries to various topics, this time sorting by ‘date’ instead of ‘relevance’ to identify new or updated content.

### Dissemination & sustainability

Our team has worked to promote the VL with collaborative partners and related organizations within Nepal and the US, such as the Nepal Health Research Council (NHRC) [47], the U.S. National Institutes of Health (NIH) Fogarty International Center [48], Two Worlds Cancer Collaboration [49], and the UVA Center for Global Health Equity [50]. We aim to longitudinally track user and website traffic metrics (including the global location from which users access the website) using Google Analytics, feedback related to specific resources, and user suggestions to enhance content and promotion and awareness of the VL. To ensure sustainability, a subset of the original VL team (10 members; 6 from Nepal, 4 from UVA) volunteered to be part of the ‘VL Sustainability Group.’ Beginning in February 2022, the VL Sustainability Group has been meeting monthly over Zoom to discuss future directions and goals of the VL. Specific focal areas for the VL Sustainability Group include ensuring current content is updated; curating new content; tracking and analyzing webpage metrics; adding enhanced features and functionality, such as a way for users to archive favorite resources and communicate with other VL users; and strategies to promote visibility of the VL. The ultimate goal is that the VL will transition, with support from UVA, from a jointly-managed project to one autonomously maintained by colleagues within Nepal. We recognize that for the VL to remain viable over the long term, we need commitment on an organizational level, such as from NAPCare or NHRC, and we are actively working to ensure this engagement.

## Discussion

This HRCB project is distinctive because of our systematic, collaborative approach to creating a web-based repository using a replicable search strategy and the RSG to assess the quality of resources. The large majority (over 76%) of resources screened using the RSG met the quality criteria of relevant, reputable, accessible, and understandable. This was somewhat surprising, as we hypothesized this percentage would be lower. We wrongly assumed many resources would score low on ‘relevance’ for not being geared towards novice researchers in an LMIC context. However, in hindsight, it is perhaps not surprising the scores were higher than expected due to the fact that best practices and core principles related to health research (e.g. study designs; ethical considerations regarding human subject research) are universally relevant. Although we attempted to account for cultural and contextual relevance of each screened resource, it proved difficult to comprehensively operationalize these concepts with the RSG. Future work should explore ways to more precisely assess the nuance of relevance and usefulness of electronic research-related resources across cultural contexts. For example, resources may be more or less relevant in Nepal if they are presented in the Nepali language or use HIC-specific examples (e.g. in Nepal, NHRC regulations are more relevant than NIH’s).

It is possible that reviewed resources were found to be highly reputable and accessible due to our conscientious search strategy, which included reputable web domains, and searching only open-access databases. While RSG scores suggest that most resources were rated by reviewers as ‘easy’ or ‘very easy’ to review and subjectively as ‘good’, team members noted during debriefing that our emphasis on open-access resources may have omitted some well-known high-quality resources simply because they were not available due to cost, copyright or licensing.

It is noteworthy that about 25% (75 of 298) of resources that were ultimately published on the VL were identified not through our systematic searching and evaluation, but rather because team members relied upon their pre-existing knowledge of well-known ‘gold-standard’ health research resources. While this was not an a priori search strategy, these team-member identified resources were still evaluated using the RSG and were subject to the same peer review process before final selection and publication on the VL. This suggests that while the RSG was essential to systematically structure our search for high-quality resources – and provide a mechanism for reproducibility – team member expertise was also a valuable source of content. Adding subjectivity and flexibility to the process – such as modifying search queries, adding Google in addition to GCS, and consulting with expert colleagues – was sometimes needed in order to refine the process and fill in obvious gaps.

It is worth re-emphasizing that a major strength of this project was the collaborative synergy between interprofessional team members and the advantage of bilateral expertise from both a LMIC and a HIC. Ultimately, the project’s success was directly related to a commendable level of commitment and engagement by all team members, which is especially noteworthy given the significant disruption caused by the COVID-19 global pandemic. We found that pairing team members from Nepal and the US during the peer-review process created an unexpected opportunity for bi-directional knowledge sharing about health research topics, and was one of the most successful, rewarding aspects of the project. We also found that search results varied by geographic setting – for example, Nepalese team members found videos in Hindi or content produced by India-based organizations, whereas UVA team member searches resulted in content created by US-based universities and organizations, like the NIH. This leveraged perspectives from both low- and high-resource contexts about resource relevance and usefulness. For future work, we would recommend even earlier bilateral peer engagement, for example by starting with LMIC-HIC partnered searches and resource evaluation for the same topic. To ensure rigor, we engaged in continuous debriefing and member checking [51]. We found it extremely helpful to re-watch recordings of Zoom meetings in which we discussed data interpretation and manuscript development.

### Limitations

The VL does not include copyrighted resources, or content behind paywalls. While this may exclude some important resources, our priority was to ensure that all content is freely available to eliminate cost barriers. After in-depth discussions about language accessibility for a global audience, noting that English is the primary language for clinical care and scientific research in Nepal and other LMICs, we published the VL in English. We included a plug-in translation feature (GTranslate [52]) which allows users to toggle between English and Nepali, with the option to add other languages later. Additionally, despite our best efforts to ensure a rigorous and comprehensive screening process, it is likely some important resources were missed. We attempted to mitigate this by including a feature that allows users to submit suggestions for other resources; we see the VL as a dynamic enterprise which will continue to evolve and iteratively be refined. Finally, we acknowledge that the content selection may have been biased by the professional expertise and country of origin of our team members, and our focus on clinical research. While we found high levels of quality across four domains, we recognize that the content of the VL may not be generalizable to settings not represented by the perspectives on our team.

## Conclusion

This paper describes the process of co-creating a ‘Virtual Library,’ a web-based repository of research resources to support investigators in LMICs and resource-limited settings. Our global team included partners from Nepal and the US and exemplifies the continuation and growth of a 17+ year-long collaboration. The development process of the VL involved the systematic review of electronic resources using a customized screening guide that prioritized relevance to the LMIC context; reputable sources and creators; free accessibility; and understandability for novice researchers. The published VL reflects a highly collaborative global partnership and offers a model for future HRCB efforts. We invite the journal’s readership to use and engage with the Virtual Library <https://lmicresearch.org> as one pillar of infrastructure to support individual and institutional research capacity.

## Supplementary Material

Supplemental Material

## Data Availability

De-identified, aggregated data are available from the corresponding author upon reasonable request and in compliance with institutional data sharing protocols.

## Paper context

Research capacity of individuals and institutions in limited-resource contexts can be strengthened through the use of web-based repositories of context-relevant resources. Our global team engaged in bi-directional peer-review to create a replicable search strategy and standardized screening guide to systematically identify high-quality resources for a ‘Virtual Library”, organized by stages of the research process. Our process represents an effective model for global, participatory health research. We invite engagement with the ‘Virtual Library’ https://lmicresearch.org
