# Supplementary material for: Building a ‘Virtual Library’: continuing a global collaboration to strengthen research capacity within Nepal and other low- and middle-income countries
Source: Glob Health Action. 2022 Oct 6;15(1):2112415. doi: 10.1080/16549716.2022.2112415 (PMC9553149; doi:10.1080/16549716.2022.2112415)
Supplement: Supplemental Material [file ZGHA_A_2112415_SM2493.docx]

**Online Supplementary Material**

**Table 6. Virtual Library Web-page Template**

| **Topic/Sub-topic:** Type your topic here.  **Your Name: Type your name.**  **Date:** Month, Day Year [e.g., January 20, 2021]  **Key Points**  · Summarize 2-3 key points about your topic using bullet points. Use simple, clear language and focus on big concepts. Be sure to phrase it in your own words and not “cut and paste.”  · Key point 2  · Key point 3  **Helpful Links, Videos, and On-Line Courses** **Helpful Overview** *(what you consider the ‘best’ overview(s) of your topic.)* · [Embed a link](http://www.google.com/) (To do this: highlight the words where you want the link to be, right click, click on “Link” or “Hyperlink” and then paste the link and click on OK.)  · For each resource you can provide a very brief description to help the user  · For each resource you can provide a very brief description to help the user **Helpful Videos** *(If nothing applies, delete heading.)* · [Embed a link](http://www.google.com/) (To do this: highlight the words where you want the link to be, right click, click on “Hyperlink” and then paste the link and click on OK.)  · For each resource you can provide a very brief description to help the user  · For each resource you can provide a very brief description to help the user **On-Line Courses** *(If nothing applies, delete heading.)* · [Embed a link](http://www.google.com/) (To do this: highlight the words where you want the link to be, right click, click on “Hyperlink” and then paste the link and click on OK.)  · For each resource you can provide a very brief description to help the user  · For each resource you can provide a very brief description to help the user  **Templates and Examples** *(If nothing applies, delete heading.)*  · This can also be a bulleted list  · For each resource you can provide a very brief description to help the user  · You can also [embed links](http://www.google.com/).  **Relevant Publications** *(If nothing applies, delete heading.)*  · This can also be a bulleted list. Articles ideally should be within last 5 years.  · You can embed the link to the article. Article should be open-access.  · Please use consistent APA citation style for references. [You can get examples or have the app generate your references here.](https://www.scribbr.com/apa-citation-generator/) |
| --- |

**Table 7. Final Formatting Checklist**

| **Virtual Library - Final Formatting Checklist**  ***Please ensure:***    Spell checked / corrected ____  Grammar checked / corrected _____    All hyperlinks embedded _____    All hyperlinks functional and open-access _____  References in APA format ______    · *Example of journal article in APA format:*  Drollinger, T., Comer, L. B., & Warrington, P. T. (2006). Development and validation of the active empathetic listening scale. *Psychology & Marketing, 23*(2), 161-180.    Font size for body text [non-headings] 12 point font _____    Left justify all text ______  Any extraneous text/comments/instructions removed ______    ***Then please:***    Re-save as word document and upload into “Ready For Final Check Folder” for the website developer. |
| --- |
